# Tuberculosis in children

**DOI:** 10.1186/1824-7288-40-S1-A4

**Published:** 2014-08-11

**Authors:** Susanna Esposito, Nicola Principi

**Affiliations:** 1 Pediatric Highly Intensive Care Unit, Department of Pathophysiology and Transplantation, Università degli Studi di Milano, Fondazione IRCCS Ca’ Granda Ospedale Maggiore Policlinico, Milan, Italy

## 

The impact of tuberculosis (TB) worldwide remains a serious concern with an estimated 8.7 million new cases (13% co-infected with HIV) and 1.4 million deaths due to TB (430,000 in HIV-infected individuals) in 2011. Assessing the impact of TB in children is particularly challenging since there is no universal diagnostic algorithm. The identification of TB cases in children usually results from a combination of clinical criteria and a non-specific TB test. In 2011, an estimated 490,000 TB cases occurred among children (about 6% of the all cases). Each year, 64,000 children die from TB, making it one of the top ten causes of childhood death.

The global burden of childhood TB is under-reported due to paucibacillary disease which makes diagnosis by sputum smear microscopy and culture difficult. In 2007, the World Health Organization showed that smear-positive TB in children accounted for 0.6-3.6% of reported cases. These data underestimate the true burden of pediatric TB since incidence is estimated using smear-positive cases. The majority of cases in children less than 12 years of age are smear-negative, and smears are seldom performed in high-burden countries. In low-burden countries, childhood TB constitutes about 5% of TB cases compared to the 20-40% in high-burden countries.

Multidrug-resistant TB (MDR-TB) is another important issue for childhood TB that affects global TB control. The global estimate of pediatric MDR-TB is around 40,000 cases per year. Almost 60% of these cases were in India, China, and Russia. The drugs recommended for these cases are off-label for children. Thus, MDR-TB is also a problem for children in close contact with adults who have MDR-TB.

Despite undeniable advances in identifying markers of TB in recent years, many problems pediatricians face in managing TB remain unsolved. The most important difficulty lies in early diagnosis because treatment can completely cure the majority of cases where TB is suspected early. Achieving a cure is more difficult when treatment is delayed and when MDR pathogens are the cause of the disease. In these cases, prognosis is poor, particularly in children, because what can be done to treat MDR-TB is unclear. New studies of diagnostic tests and optimal treatment for children are urgently needed with the final goal of developing an effective anti-TB vaccine. In the meantime, an aggressive attitude must be adopted for both diagnosing and treating a child with suspected TB because TB can be a devastating disease for children.

